# Comparing treatment and outcomes in advanced esophageal, gastroesophageal junction, and gastric adenocarcinomas: a population-based study

**DOI:** 10.1177/17588359231162576

**Published:** 2023-03-21

**Authors:** Marieke Pape, Pauline A. J. Vissers, Willemieke P. M. Dijksterhuis, David Bertwistle, Laura McDonald, Bianca Mostert, Sarah Derks, Irma M. Oving, Rob H. A. Verhoeven, Hanneke W. M. van Laarhoven

**Affiliations:** Department of Research & Development, Netherlands Comprehensive Cancer Organisation (IKNL), Utrecht, the Netherlands; Amsterdam UMC location University of Amsterdam, Medical Oncology, Amsterdam, the Netherlands; Cancer Center Amsterdam, Cancer Treatment and Quality of Life, Amsterdam, the Netherlands; Department of Research & Development, Netherlands Comprehensive Cancer Organisation (IKNL), Utrecht, the Netherlands; Department of Surgery, Radboud University Medical Centre, Nijmegen, the Netherlands; Department of Research & Development, Netherlands Comprehensive Cancer Organisation (IKNL), Utrecht, the Netherlands; Amsterdam UMC location University of Amsterdam, Medical Oncology, Amsterdam, the Netherlands; Cancer Center Amsterdam, Cancer Treatment and Quality of Life, Amsterdam, the Netherlands; Worldwide Health Economics & Outcomes Research, Bristol-Myers Squibb, Uxbridge, UK; Worldwide Health Economics & Outcomes Research, Bristol-Myers Squibb, Uxbridge, UK; Department of Medical Oncology, Erasmus MC Cancer Institute, Rotterdam, the Netherlands; Amsterdam UMC location Vrije Universiteit Amsterdam, Medical Oncology, Amsterdam, the Netherlands; Cancer Center Amsterdam, Cancer Biology and Immunology, Amsterdam, the Netherlands; Oncode Institute, Utrecht, The Netherlands; Department of Medical Oncology, Ziekenhuisgroep Twente, Almelo, the Netherlands; Department of Research & Development, Netherlands Comprehensive Cancer Organisation (IKNL), Utrecht, the Netherlands; Amsterdam UMC location University of Amsterdam, Medical Oncology, Amsterdam, the Netherlands; Cancer Center Amsterdam, Cancer Treatment and Quality of Life, Amsterdam, the Netherlands; Amsterdam UMC location University of Amsterdam, Medical Oncology, Meibergdreef 9, Amsterdam 1105 AZ, the Netherlands; Cancer Center Amsterdam, Cancer Treatment and Quality of Life, Amsterdam, the Netherlands

**Keywords:** esophageal adenocarcinoma, gastric adenocarcinoma, gastroesophageal junction adenocarcinoma, overall survival, systemic therapy

## Abstract

**Background:**

Treatment of advanced or metastatic esophageal adenocarcinoma (EAC) follows the guidelines for gastroesophageal junction adenocarcinoma (GEJC) and gastric adenocarcinoma (GAC), but patients with EAC are often excluded from clinical studies of GEJC/GAC.

**Objectives:**

Here we describe treatment and survival of patients with advanced EAC, GEJC, and GAC to provide population-based evidence on distinctions and similarities between these populations.

**Design:**

Retrospective cohort study of patients with unresectable advanced (cT4b) or metastatic (cM1) EAC, GEJC, or GAC (2015–2020) were selected from the Netherlands Cancer Registry.

**Methods:**

Overall survival (OS) was assessed using Kaplan–Meier methods, log-rank tests, and multivariable Cox regression.

**Results:**

In all, 7391 patients were included (EAC: *n* = 3346, GEJC: *n* = 1246, and GAC: *n* = 2798). Patients with EAC were more often males and more often had ⩾2 metastatic locations. First-line systemic therapy was received by 42%, 47%, and 36% of patients with EAC, GEJC, and GAC, respectively. Median OS was 5.0, 5.1, and 4.0 months for all patients with EAC, GEJC, and GAC, respectively (*p* < 0.001). Median OS from start of first-line therapy of patients with human epidermal growth factor receptor 2 (HER2)-negative adenocarcinomas was 7.6, 7.8, and 7.5 months (*p* = 0.12) and of patients with HER2-positive carcinoma receiving first-line trastuzumab-containing therapy was 11.0, 13.3, and 9.5 months (*p* = 0.37) in EAC, GEJC, and GAC, respectively. After multivariable adjustment, no difference in OS for patients with EAC, GEJC, and GAC was observed.

**Conclusion:**

Despite differences in clinical characteristics and treatment strategies, survival between patients with advanced EAC, GEJC, and GAC was similar. We advocate that EAC patients should not be excluded from clinical trials for patients with molecularly similar GEJC/GAC.

## Introduction

Esophageal adenocarcinoma (EAC) is the most common subtype of esophageal cancer in Western countries and accounts for around two-thirds of cases.^1^ In gastric and gastroesophageal junction cancers, 95% of cases are adenocarcinoma.^2^ The main risk factors for EAC are Barrett’s esophagus, gastroesophageal reflux disease, and obesity, the latter two are also risk factors for gastroesophageal junction adenocarcinoma (GEJC), whereas the main risk factor for developing gastric adenocarcinoma (GAC) is a *Helicobacter pylori* infection.^3,4^

Gastric cancer can be classified into four distinct genomic subtypes.^5^ One such subtype is characterized by an intestinal histology and exhibits chromosomal instability (CIN).^5^ On a molecular level, EAC strongly resembles the intestinal CIN subtype as opposed to esophageal squamous cell carcinoma which is molecularly distinct from the gastric cancer genomic subtypes.^6^ EAC, GEJC, and GAC share molecular similarities and could therefore be considered the same disease entity.^6^

According to the current European Society for Medical Oncology (ESMO) guidelines for gastric cancer, first-line palliative systemic treatment consists of a fluoropyrimidine and platinum doublet, with the addition of trastuzumab in patients with a human epidermal growth factor receptor 2 (HER2)-positive tumor.^7–10^ Recently, the addition of nivolumab to first-line chemotherapy showed an improved survival compared to chemotherapy alone in patients with advanced EAC, GEJC, or GAC, particularly for programmed death-ligand 1 combined positive score ⩾5.^11^ In second line, paclitaxel in combination with ramucirumab is considered standard of care.^12^ However, evidence for the efficacy systemic therapy in patients with EAC is limited, as these patients were excluded from pivotal clinical trials for GEJC/GAC (Supplemental Table 1).^8,11–16^ Although ESMO guidelines for palliative systemic therapy advise to treat patients with EAC mainly according to the guidelines of GEJC/GAC, this may be hampered by formal registration constraints.^7,17^ For example, the EMA registration of ramucirumab is limited to GEJC/GAC due to exclusion of patients with EAC from the pivotal RAINBOW trial.^12^

In this population-based study, we compared treatment strategies, survival, and time to treatment failure (TTF) of patients with advanced EAC, GEJC, and GAC to provide population-based evidence on distinctions and similarities of these populations.

## Methods

### Study population

Patients with an adenocarcinoma of the esophagus (C15.0–C15.9), gastroesophageal junction/cardia (C16.0), or stomach (C16.1–C16.9) diagnosed with unresectable advanced (cT_4b_cN_all_cM_0_) or synchronous metastatic disease (cT_all_cN_all_cM_1_) in 2015–2020 were selected from the Netherlands Cancer Registry (NCR) (Supplemental Figure 1). Tumors were staged using the International Union Against Cancer TNM classification 7th edition (2015–2016) and 8th edition (2017–2020).^18,19^

The NCR is a nationwide population-based cancer registry that covers the Dutch population and is based on notification of all newly diagnosed malignancies by the national automated pathology archive. Registration clerks of the NCR routinely extract information on diagnosis, tumor stage, and treatment from medical records. For patients diagnosed in 2015–2017, data on TTF and second-line systemic therapy were collected in the second half of 2019 in all Dutch hospitals (except two hospitals due to logistical reasons). Information on vital status was available through linkage of the NCR with the Dutch Personal Records Database and follow-up was complete until 1 February 2022.

### HER2 status

HER2 status was classified as positive, negative, or unknown. HER2 status was classified as positive if immunohistochemistry (IHC) test result was 3+ [not followed by in situ hybridization (ISH) test] or in case of IHC test result of 1+ or 2+ followed by a positive ISH test. HER2 status was classified as negative if IHC test result of 0 or 1+ (not followed by IHC test), or if IHC test result of 2+ or 3+ was followed by a negative ISH. If there was no mentioning of HER2 testing or the results of IHC and ISH testing were inconclusive, HER2 status was classified as unknown.

### Treatment definitions

Treatment was classified into mutually exclusive groups in the following order: palliative resection (endoscopic or surgical resection of primary tumor and/or metastasectomy with or without perioperative treatment), chemoradiotherapy (chemotherapy with concurrent radiotherapy with an overlap of at least 7 days), systemic therapy, and best supportive care. A systemic treatment regimen was defined as all chemotherapy and targeted therapy agents that started within 3 days of each other, as previously described.^20,21^ Second-line therapy was considered when a new agent of a different drug group was started that was not administered in first-line.^21^ As first-line systemic treatment recommendations differ according to HER2 status, analysis for first-line therapy was stratified for patients with a HER2-positive tumor, a HER2-negative tumor, and an unknown HER2 status.

### Overall survival and TTF

For analyses of all patients and patients receiving best supportive care only, overall survival (OS) was assessed from the date of diagnosis. For analyses of patients receiving first-line systemic therapy, OS and TTF were assessed from the start of first-line treatment. OS was assessed until death or end of follow-up. TTF was assessed as previously described by Dijksterhuis *et al*.^20^ In short, TTF was assessed until the first progression that resulted in the end of first-line treatment. Patients were censored on date of the last hospital visit if no progression was registered.

### Statistical analyses

Patient, tumor, and treatment characteristics were compared between patients with EAC, GEJC, and GAC using chi-squared test, Fisher’s exact test, or ANOVA where appropriate. To evaluate OS and TTF, Kaplan–Meier methods and log-rank tests were used. OS was also evaluated using univariable and multivariable Cox proportional hazard models. The multivariable models were adjusted for available clinical characteristics. The proportional hazards assumptions for these variables were tested with time-dependent covariates as a function of the survival time. If these interaction terms were significant, the Schoenfeld residual plots were graphically inspected and if the residuals of the covariates changed over time, the covariates were deemed non-proportional and the Cox model was stratified for these covariates instead of adjusted. *p* Values of <0.05 were considered statistically significant. All analyses were conducted using SAS® version 9.4 (SAS Institute, Cary, NC, USA).

## Results

### Baseline characteristics

In total, 7390 patients were included with an adenocarcinoma located in the esophagus (*n* = 3346, 45%), gastroesophageal junction/cardia (*n* = 1246, 17%), or stomach (*n* = 2798, 38%) (Table 1). The proportion of males was 83%, 75%, and 61% for patients with EAC, GEJC, and GAC, respectively (*p* < 0.001). Median age was 68, 69, and 72 years for patients with EAC, GEJC, and GAC, respectively (*p* < 0.001). Patients with EAC more often had ⩾2 metastatic locations (50%) compared to patients with GEJC (43%) and GAC (32%). Location of metastases differed between patients with EAC, GEJC, and GAC (Table 1). Patients with EAC and GEJC less often had a diffuse (EAC: 14%, GEJC: 22%) than intestinal tumor type (EAC: 45%, GEJC: 47%) as compared to patients with GAC (diffuse: 47%, intestinal: 32%).

**Table 1.** Baseline characteristics of patients with esophageal, gastroesophageal junction or gastric cancer.

	All patients (*n* = 7390)	Esophageal (*n* = 3346)	Gastroesophageal junction (*n* = 1246)	Gastric (*n* = 2798)	*p* Value
Sex, *n* (%)					<0.0001^1^
Male	5423 (73%)	2793 (83%)	936 (75%)	1694 (61%)	
Female	1967 (27%)	553 (17%)	310 (25%)	1104 (39%)	
Age					<0.0001^2^
Median (IQR)	69 (61–77)	68 (61–75)	69 (61–76)	72 (62–78)	
Number of comorbidities, *n* (%)					0.5886^1^
0	3521 (48%)	1607 (48%)	604 (48%)	1310 (47%)	
1	2238 (30%)	1009 (30%)	378 (30%)	851 (30%)	
⩾2	1310 (18%)	599 (18%)	211 (17%)	500 (18%)	
Unknown	321 (4%)	131 (4%)	53 (4%)	137 (5%)	
ECOG performance status, *n* (%)					<0.0001^1^
0–1	3627 (49%)	1789 (53%)	652 (52%)	1186 (42%)	
⩾2	1379 (19%)	595 (18%)	240 (19%)	544 (19%)	
Unknown	2384 (32%)	962 (29%)	354 (28%)	1068 (38%)	
Type of disease, *n* (%)					<0.0001^1^
Unresectable advanced (cT4b-cM0)	233 (3%)	43 (1%)	26 (2%)	164 (6%)	
Metastatic disease (cM1)	7157 (97%)	3303 (99%)	1220 (98%)	2634 (94%)	
Tumor location, *n* (%)					<0.0001^1^
Cervical, proximal or middle esophagus	202 (3%)	202 (6%)	0 (0%)	0 (0%)	
Distal esophagus	2938 (40%)	2938 (88%)	0 (0%)	0 (0%)	
Overlapping/unknown esophagus	206 (3%)	206 (6%)	0 (0%)	0 (0%)	
Gastroesophageal junction/cardia	1246 (17%)	0 (0%)	1246 (100%)	0 (0%)	
Gastric fundus or corpus	857 (12%)	0 (0%)	0 (0%)	857 (31%)	
Gastric antrum or pylorus	864 (12%)	0 (0%)	0 (0%)	864 (31%)	
Gastric overlapping/unknown	1077 (15%)	0 (0%)	0 (0%)	1077 (38%)	
cT stage, *n* (%)					<0.0001^3^
cT1	23 (0%)	13 (0%)	4 (0%)	6 (0%)	
cT2	1576 (21%)	855 (26%)	228 (18%)	493 (18%)	
cT3	2875 (39%)	1466 (44%)	551 (44%)	858 (31%)	
cT4	955 (13%)	196 (6%)	137 (11%)	622 (22%)	
cTX	1961 (27%)	816 (24%)	326 (26%)	819 (29%)	
cN stage, *n* (%)					<0.0001^1^
cN0	1403 (19%)	364 (11%)	181 (15%)	858 (31%)	
cN1	2095 (28%)	1024 (31%)	376 (30%)	695 (25%)	
cN2	2473 (33%)	1303 (39%)	449 (36%)	721 (26%)	
cN3	754 (10%)	465 (14%)	142 (11%)	147 (5%)	
cNX	665 (9%)	190 (6%)	98 (8%)	377 (13%)	
Lauren classification, *n* (%)					<0.0001^1^
Intestinal	2985 (40%)	1518 (45%)	582 (47%)	885 (32%)	
Diffuse	2044 (28%)	460 (14%)	269 (22%)	1315 (47%)	
Mixed	224 (3%)	82 (2%)	40 (3%)	102 (4%)	
Indeterminate	234 (3%)	130 (4%)	35 (3%)	69 (2%)	
Adenocarcinoma NOS	1903 (26%)	1156 (35%)	320 (26%)	427 (15%)	
Tumor differentiation, *n* (%)					<0.0001^1^
Well/moderate	1688 (23%)	951 (28%)	325 (26%)	412 (15%)	
Poorly/undifferentiated	2905 (39%)	1249 (37%)	495 (40%)	1161 (41%)	
Unknown	2797 (38%)	1146 (34%)	426 (34%)	1225 (44%)	
HER2 status^4^, *n* (%)					<0.0001^1^
Positive	974 (13%)	562 (17%)	189 (15%)	223 (8%)	
Negative	3620 (49%)	1492 (45%)	677 (54%)	1451 (52%)	
Unknown	2796 (38%)	1292 (39%)	380 (30%)	1124 (40%)	
Distant metastatic sites, *n* (%)					<0.0001^1^
0	233 (3%)	43 (1%)	26 (2%)	164 (6%)	
1	4050 (55%)	1614 (48%)	684 (55%)	1752 (63%)	
⩾2	3107 (42%)	1689 (50%)	536 (43%)	882 (32%)	
Non-regional lymph nodes metastases, *n* (%)	2934 (40%)	1615 (48%)	534 (43%)	785 (28%)	<0.0001^1^
Lung metastases, *n* (%)	1261 (17%)	828 (25%)	190 (15%)	243 (9%)	<0.0001^1^
Liver metastases, *n* (%)	3298 (45%)	1804 (54%)	648 (52%)	846 (30%)	<0.0001^1^
Peritoneal metastases, *n* (%)	2140 (29%)	262 (8%)	321 (26%)	1557 (56%)	<0.0001^1^
Bone metastases, *n* (%)	1104 (15%)	751 (22%)	139 (11%)	214 (8%)	<0.0001^1^
Other metastatic sites, *n* (%)	1028 (14%)	614 (18%)	154 (12%)	260 (9%)	<0.0001^1^

1 Chi-square *p* value.

2 ANOVA *p* value.

3 Fisher’s exact *p* value.

4 Unknown HER2 status includes patients for who HER2 status was not tested or inconclusive after testing.

ANOVA, analysis of variance; ECOG, Eastern Cooperative Oncology Group; HER2, human epidermal growth factor receptor 2; IQR, interquartile range.

### Treatment patterns

**Figure 1.**
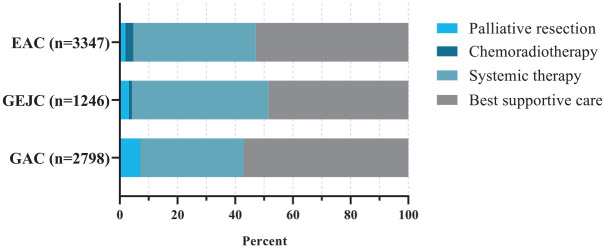
Type of treatment in patients with EAC, GEJC, or GAC. Treatment groups were classified into four mutually exclusive groups in the following order: palliative resection (with or without perioperative treatment), chemoradiotherapy, systemic therapy, and best supportive care. EAC, esophageal adenocarcinoma; GAC, gastric adenocarcinoma; GEJC, gastroesophageal junction adenocarcinoma.

Type of treatment was statistically significantly different between patients with EAC, GEJC, and GAC (*p* < 0.001; Figure 1). The percentage of patients receiving first-line systemic therapy (without resection or radiation) was 42%, 47%, and 36% for EAC, GEJC, and GAC, respectively. A small proportion of patients underwent palliative resection: 2%, 3%, and 7% in EAC, GEJC, and GAC, respectively. Chemoradiotherapy was administered to 3%, 1%, and <1% of patients with EAC, GEJC, and GAC, respectively. Best supportive care was received by 53%, 48%, and 57% of patients with EAC, GEJC, and GAC, respectively.

**Figure 2.**
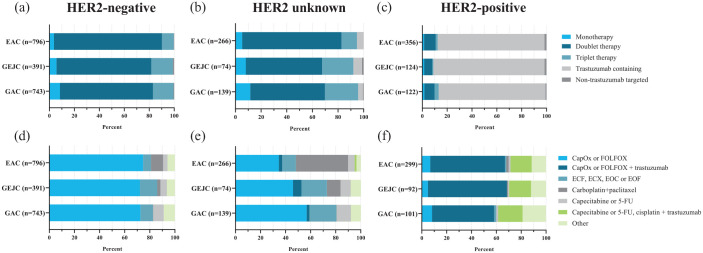
Type of first-line systemic treatment in patients with EAC, GEJC, or GAC stratified by HER2 status. The top three graphs present the general classification and the bottom three graphs present the classification by specific regimens if occurring in at least 100 patients. Patients who received palliative resection or chemoradiotherapy (chemotherapy with concurrent radiotherapy independent of dosage with an overlap of at least 7 days) were not included. 5-FU, 5-fluorouracil, CapOx, capecitabine and oxaliplatin; EAC, esophageal adenocarcinoma; ECF, epirubicin, cisplatin, and 5-FU; ECX, epirubicin, cisplatin, and capecitabine; EOF, epirubicin, oxaliplatin, and 5-FU; EOX, epirubicin, oxaliplatin, and capecitabine; GAC, gastric adenocarcinoma; GEJC, gastroesophageal junction adenocarcinoma; FOLFOX, 5-FU and oxaliplatin; HER2, human epidermal growth factor receptor 2.

Among patients with HER2-negative cancer who received first-line systemic therapy, doublet therapy was received by 87%, 76%, and 75% for EAC, GEJC, and GAC, respectively (Figure 2). Most common doublet therapy was capecitabine or 5-fluorouracil (5-FU) and oxaliplatin [capecitabine and oxaliplatin (CapOx)/5-FU and oxaliplatin (FOLFOX)], in 73%, 72%, and 75% of patients with EAC, GEJC, and GAC, respectively. Patients with HER2-negative EAC more often received carboplatin and paclitaxel (without resection or radiation) (10%) compared to patients with GEJC (3%) and GAC (<1%). Among patients with an unknown HER2 status, the administration of carboplatin and paclitaxel was higher (42%) in EAC compared to GEJC (11%) and GAC (0%). Among patients with HER2-positive cancer, a trastuzumab-containing regimen was administered to 86%, 90%, and 86% of EAC, GEJC, and GAC, respectively.

Among patients diagnosed in 2015–2017 who received first-line therapy and for whom complete follow-up was available (*n* = 1324), second-line therapy was administered to 393 patients (30%). Patients with EAC less often received second-line paclitaxel and ramucirumab (35%) compared to patients with GEJC (60%) and GAC (66%) (*p* < 0.001; Supplemental Figure 2). None of the patients who received first-line carboplatin and paclitaxel received second-line paclitaxel and ramucirumab (Supplemental Figure 3). Most common second-line regimens in patients who received first-line carboplatin and paclitaxel were CapOx/FOLFOX (37%) and 5-FU and irinotecan (18%).

### Survival

**Figure 3.**
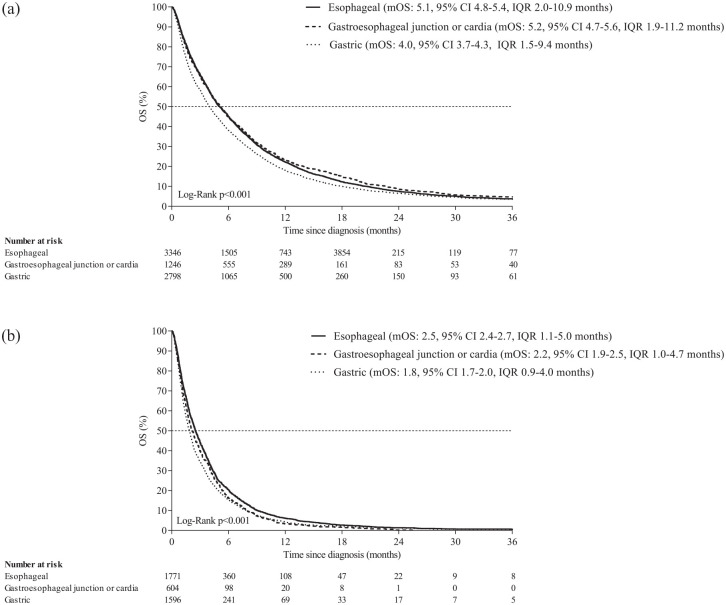
OS of patients with EAC, GEJC or GAC independent of treatment (a), and those receiving best supportive care (b). EAC, esophageal adenocarcinoma; GAC, gastric adenocarcinoma; GEJC, gastroesophageal junction adenocarcinoma; IQR, interquartile range; mOS, median OS; OS, overall survival.

Median OS for all patients was 4.6 months, 1- and 3-year survival rates were 21% and 4%, respectively (Supplemental Table 2). Median OS was 5.1, 5.2, and 4.0 months for patients with EAC, GEJC, and GAC (*p* < 0.001; Figure 3(a)). Among patients with intestinal tumor type, median OS was 5.9, 5.6, and 4.2 for EAC, GEJC, and GAC, respectively (*p* = 0.001; data not shown). Among patients with EAC, GEJC, and GAC receiving best supportive care, median OS was 2.5, 2.2, and 1.8 months, respectively (*p* < 0.001; Figure 3(b)).

**Figure 4.**
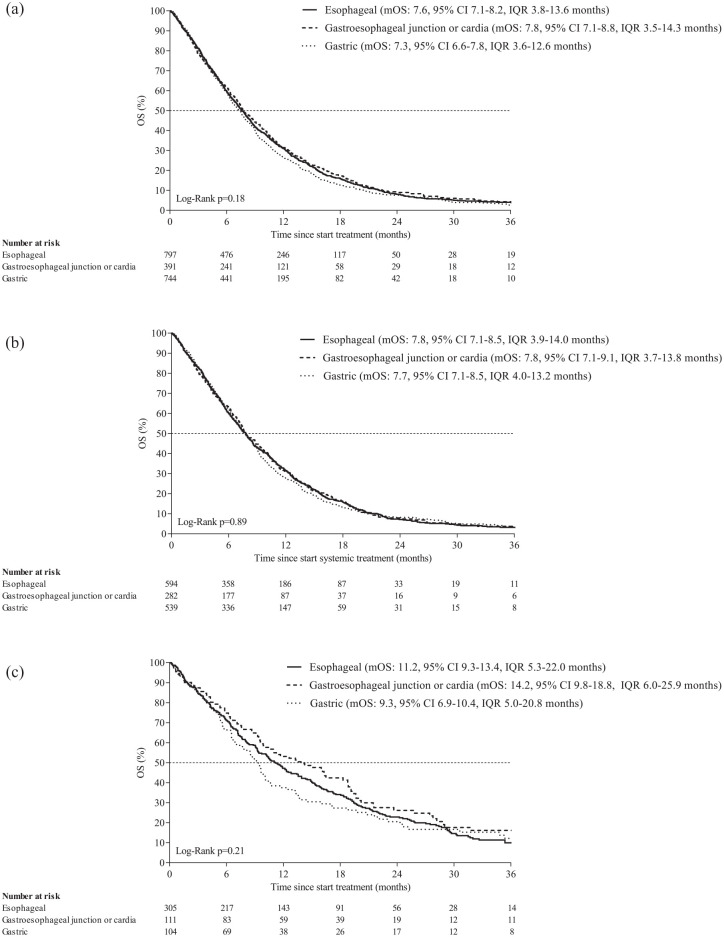
OS of patients with esophageal, gastroesophageal junction, or gastric HER2-negative cancer receiving any first-line treatment (a), receiving first-line capecitabine or 5-FU with oxaliplatin (b), and receiving a trastuzumab containing regimen (c). IQR, interquartile range; mOS, median OS, OS, overall survival.

Among patients with HER2-negative cancer receiving first-line therapy, median OS was 7.6, 7.8, and 7.3 months for EAC, GEJC, and GAC (*p* = 0.18; Figure 4(a)). Among patients with HER2-negative cancer receiving first-line CapOx/FOLFOX, median OS was 7.8, 7.8, and 7.7 months for EAC, GEJC, and GAC, respectively (*p* = 0.89; Figure 4(b)). Among patients with a performance status of 0–1 receiving HER2-negative cancer receiving first-line CapOx/FOLFOX, median OS was 8.3, 8.8, and 8.6 months for EAC, GEJC, and GAC, respectively (*p* = 0.87; Supplemental Figure 5). Among patients with HER2-negative EAC receiving first-line carboplatin and paclitaxel (*n* = 77), median OS was 8.8 months (data not shown). Among patients with HER2-positive cancer receiving a trastuzumab containing regimen in first line, median OS was 11.2, 14.2, and 9.3 months for EAC, GEJC, and GAC, respectively (*p* = 0.21; Figure 4(c)). Results of TTF analyses in patients with complete follow-up are provided in Supplemental Table 3.

Among patients receiving second-line systemic therapy, median OS from start of second-line treatment was 5.5, 6.4, and 5.3 months for EAC, GEJC, and GAC, respectively (*p* = 0.78; Supplemental Figure 4A). Among patients receiving second-line paclitaxel and ramucirumab, median OS was 6.5, 7.3, and 5.6 months for EAC, GEJC, and GAC (*p* = 0.18; Supplemental Figure 4B).

Compared to patients with EAC, multivariable Cox regression analysis showed no differences in survival between patients with GEJC [hazard ratio (HR): 1.07, 95% confidence interval (CI): 0.99–1.15] and GAC (HR: 1.05, 95% CI: 0.98–1.13) (Table 2; Supplemental Table 4). Among patients with an intestinal tumor type, multivariable analysis showed no difference in survival between GEJC (HR: 1.07, 95% CI: 0.96–1.20) and GAC (HR 1.03, 95% CI 0.91–1.15) compared to EAC. Similarly, for patients receiving best supportive care no differences between the three groups were observed. Multivariable analysis for patients with HER2-negative cancer receiving first-line therapy and for patients with HER2-positive cancer receiving a trastuzumab containing regimen in first-line showed no difference in survival between the three groups.

**Table 2.** Multivariable Cox regression of OS in patients with esophageal, gastroesophageal junction, or gastric cancer.

	Number of patients	Events	Median OS (months)^a^	Univariable regression, HR (95% CI)	Multivariable regression, HR (95% CI)^b,c^
All patients^d^					
Esophageal	3346	3192	5.1	Reference	Reference
Gastroesophageal junction or cardia	1246	1178	5.2	0.97 (0.91–1.04)	1.07 (0.99–1.15)
Gastric	2798	2681	4.0	1.14 (1.09–1.20)	1.05 (0.98–1.13)
Patients with intestinal tumor type					
Esophageal	1518	1439	5.9	Reference	Reference
Gastroesophageal junction or cardia	582	543	5.6	0.98 (0.88–1.08)	1.07 (0.96–1.20)
Gastric	885	841	4.2	1.15 (1.06–1.25)	1.03 (0.91–1.15)
Patients receiving best supportive care^e^					
Esophageal	1771	1761	2.5	Reference	Reference
Gastroesophageal junction or cardia	604	602	2.2	1.13 (1.03–1.24)	0.91 (0.83–1.00)
Gastric	1596	1584	1.8	1.20 (1.12–1.28)	1.00 (0.90–1.11)
Patients with HER2-negative carcinoma receiving first-line therapy					
Esophageal	797	749	7.6	Reference	Reference
Gastroesophageal junction or cardia	391	367	7.8	0.97 (0.86–1.10)	0.98 (0.85–1.13)
Gastric	744	700	7.3	1.08 (0.97–1.19)	1.01 (0.88–1.16)
Patients with HER2-positive carcinoma receiving first-line trastuzumab containing therapy					
Esophageal	305	257	11.2	Reference	Reference
Gastroesophageal junction or cardia	111	91	14.2	0.89 (0.70–1.13)	0.87 (0.63–1.25)
Gastric	104	93	9.3	1.15 (0.91–1.46)	0.79 (0.60–1.05)

a For all patients, patients with intestinal tumor type and patients receiving best supportive care the median OS was calculated since diagnosis and for patients receiving first-line therapy since start of first-line therapy.

b Adjusted for sex, age, number of comorbidities, Lauren classification, tumor differentiation, number of metastatic sites, location of metastases (distant lymph nodes, liver metastases, lung, bone, peritoneal, and other), and type of treatment (if applicable).

c Performance status, cT-stage, cN-stage, and HER2 status did not meet the proportional hazard assumptions and the models were stratified for this covariate.

d HRs of the adjusted variables in the multivariable analysis of all patients with esophageal, gastroesophageal junction, or gastric cancer are available in Supplemental Table 3.

e Multivariable model for patients receiving best supportive care was additionally corrected for receiving radiotherapy for symptom control.

CI, confidence interval; HER2, human epidermal growth factor receptor 2; HR, hazard ratio; OS, overall survival.

## Discussion

In this nationwide population-based cohort study of patients with advanced EAC, GEJC, and GAC, we found that despite differences in patient and tumor characteristics as well as treatment, survival of patients with EAC was similar to patients with GEJC and GAC. This observation is consistent with response to cancer treatment being influenced more by the genetic profile of gastroesophageal tumors than by their anatomical location.

Recent research efforts in gastroesophageal cancers have led to the conclusion that on a molecular level EAC clusters with adenocarcinomas of the gastroesophageal junction and the CIN subtype of gastric cancer.^5,6^ The CIN subtype echoes the intestinal tumor type on a histological level.^5^ In our analysis of patients with an intestinal tumor type, also no survival differences between EAC, GEJC, and GAC were observed. In the curative setting, the four distinct genomic subtypes of gastric cancer, including the CIN subtype, have previously been associated with different prognostic outcomes.^22^ In the palliative setting, the association of the subtypes of gastric cancer with prognostic outcomes is not yet know and in the current study the association of the four genomic subtypes of gastric cancer was unavailable due to the irrelevance in daily clinical practice.

Our results are in line with a previous pooled analysis study including patients from four randomized controlled trials reported no difference in survival, objective tumor response, and toxic effects between advanced EAC, GEJC, and GAC.^23^ In addition, a retrospective observational study in the United States including patients with HER2-negative cancer receiving first-line treatment no difference in survival was reported between unresectable advanced gastric or gastroesophageal junction cancer *versus* EAC.^24^ Survival in our study among patients with performance status of 0–1 receiving CapOx/FOLFOX for a HER2-negative tumor (mOS: 8.3–8.8 months) was lower as compared to the recent phase III trial, CheckMate 649, which reported a median OS of 11.6 months for all randomly assigned patients receiving chemotherapy alone.^11^ This difference in survival is probably the result of strict exclusion criteria besides performance status of 0–1, such as the presence of other (auto-immune) diseases and/or comorbidities.

Treatment strategies differ between patients with EAC, GEJC, and GAC. In our study, we observed that patients with GAC (7%) more often underwent palliative resection as compared to EAC (2%) or GEJC (3%). Indeed, palliative gastrectomy is regarded as a treatment option to relieve symptoms, whereas an esophagectomy is not.^7,17,25,26^ The percentage of patients receiving first-line systemic therapy (without resection or radiation) was lowest in GAC (36%) compared to EAC (42%) and GEJC (47%). This could be due to several reasons: patients with GAC more often received palliative resection (with or without perioperative therapy), they were slightly older, more often female, more often had a performance status of >1, and may therefore have refrained from palliative systemic therapy.

In contrast to the ESMO guidelines primarily recommending first-line doublet therapy consisting of a fluoropyrimidine and platinum, we found a high number of EAC patients with HER2-negative or unknown HER2 status who received first-line carboplatin and paclitaxel as compared to GEJC and GAC.^7,17^ In a retrospective observational study from the United States, administration of first-line carboplatin and paclitaxel was approximately 2–5% and in a population-based study in Canada, the use of carboplatin/cisplatin and paclitaxel was 2%.^27,28^ The high proportion of patients receiving first-line carboplatin and paclitaxel appears to be clinical practice in a selection of Dutch hospitals (data not shown). This could be the result from a Dutch phase I trial that found carboplatin and paclitaxel to be well-tolerated and effective in patients with advanced esophageal cancer, and from a Dutch phase II trial which showed that capecitabine and oxaliplatin in patients with advanced or metastatic esophageal cancer in which toxicity was not less compared to carboplatin and paclitaxel.^29,30^ Another reason could be the usage of carboplatin and paclitaxel in the curative setting according to the Dutch CROSS trial.^31^

Less than a third of patients who received first-line treatment received second-line treatment. We did not observe OS differences between patients with EAC, GEJC, and GAC receiving second-line treatment. Previous studies reported a survival benefit of second-line treatment with paclitaxel and ramucirumab compared to taxane monotherapy.^12,21^ In patients with EAC, second-line paclitaxel and ramucirumab usage was almost 30% lower as compared to patients with GEJC or GAC. Patients with EAC were not eligible for the pivotal clinical trial of paclitaxel and ramucirumab in second line (RAINBOW) and consequently this regimen is not formally registered for EAC.^12^ Therefore, carboplatin and paclitaxel can be chosen in first line when paclitaxel and ramucirumab is not considered an option in second line. However, our study shows that in clinical practice patients with EAC are tested for HER2 and if positive receive trastuzumab, and second-line paclitaxel and ramucirumab. Due to the changing treatment landscape in esophagogastric cancer, further studies should investigate whether the effectiveness of novel therapies is also similar for EAC, GEJC, or GAC.

The strength of our study is the use of population-based data, which reflects treatment and outcomes including frail patients, elderly and patients with comorbidities. Our study also has limitations. First, not all patients with HER2-positive carcinoma received a trastuzumab-containing regimen, these patients (*n* = 80) were excluded from the survival analysis. It was previously reported in a population-based study from the Netherlands that 23% of patients with HER2-positive cancers did not receive a trastuzumab containing regimen which could be partly due to the lack of registration for true EAC tumors but is potentially related to cardiac comorbidity.^32^ Second, missing data on HER2 status and other variables, for example, performance status, could have resulted in suboptimal adjustment of multivariable models. Third, for patients with a HER2-positive tumor, it was unknown if the result of the IHC staining patterns was 2+/3+ which could also have contributed to suboptimal adjustment. Lastly, misclassification of the primary tumor location could have occurred as information on tumor margins was unavailable. Classification of the primary tumor was based on the diagnosis in the clinical practice.

In conclusion, we have shown that despite differences in patient and tumor characteristics, survival is comparable between patients with advanced EAC, GEJC, and GAC. We advocate that patients with EAC should not be considered a separate entity and should be included in trials encompassing molecularly similar gastroesophageal junction and gastric cancers to be able to have similar benefit from novel treatment options.

## Supplemental Material

sj-docx-1-tam-10.1177_17588359231162576 – Supplemental material for Comparing treatment and outcomes in advanced esophageal, gastroesophageal junction, and gastric adenocarcinomas: a population-based study Click here for additional data file. Supplemental material, sj-docx-1-tam-10.1177_17588359231162576 for Comparing treatment and outcomes in advanced esophageal, gastroesophageal junction, and gastric adenocarcinomas: a population-based study by Marieke Pape, Pauline A. J. Vissers, Willemieke P. M. Dijksterhuis, David Bertwistle, Laura McDonald, Bianca Mostert, Sarah Derks, Irma M. Oving, Rob H. A. Verhoeven and Hanneke W. M. van Laarhoven in Therapeutic Advances in Medical Oncology

## Declarations

**Ethics approval and consent to participate:** According to the Central Committee on Research involving Human Subjects, this type of study does not require approval from an ethics committee in the Netherlands. This study was approved by the Privacy Review Board of the NCR and the scientific committee of the Dutch Upper GI Cancer Group. Based on current Dutch legislation, it is not necessary to retrieve informed consent from patients for registration into the NCR. The privacy review board of the NCR reviews all data requests for studies with data of the NCR regarding privacy issues.

**Consent for publication:** Not applicable.

**Availability of data and materials:** The data underlying this article are available at the Netherlands Comprehensive Cancer Organisation (IKNL) upon justified request.
