# Supplementary material for: Comparing treatment and outcomes in advanced esophageal, gastroesophageal junction, and gastric adenocarcinomas: a population-based study
Source: Ther Adv Med Oncol. 2023 Mar 21;15:17588359231162576. doi: 10.1177/17588359231162576 (PMC10031599; doi:10.1177/17588359231162576)
Supplement: sj-docx-1-tam-10.1177_17588359231162576 – Supplemental material for Comparing treatment and outcomes in advanced esophageal, gastroesophageal junction, and gastric adenocarcinomas: a population-based study [file sj-docx-1-tam-10.1177_17588359231162576.docx]

**Supplementary table 1. Overview of randomized clinical trials (phase III) for gastric adenocarcionoma.**

| Study | Treatment line | Treatments | | Main inclusion criteria | Inclusion of esophageal adenocarcinoma? |
| --- | --- | --- | --- | --- | --- |
|  |  | Control group | Experimental group |  |  |
| ToGA^1^ | First-line | Capecitabine or fluorouracil plus cisplatin | Capecitabine or fluorouracil, cisplatin and trastuzumab | Inoperable locally advanced, recurrent, or metastatic adenocarcinoma of the stomach or gastro-oesophageal junction | No |
| REAL3^2^ | First-line | Epirubicin, oxaliplatin, and capecitabine | Epirubicin, oxaliplatin, and capecitabine with or without panitumumab | Metastatic or locally advanced inoperable adenocarcinoma or undifferentiated carcinoma of the oesophagus, gastro-oesophageal junction, or stomach | Yes |
| REGARD^3^ | Second-line | Placebo | Ramucirumab monotherapy | Metastatic or unresectable, locally recurrent gastric or gastro-oesophageal junction adenocarcinoma | No |
| RAINBOW^4^ | Second-line | Paclitaxel plus placebo | Paclitaxel plus ramucirumab | Metastatic or non-resectable, locally advanced gastric or gastro-oesophageal junction adenocarcinoma | No |
| KEYNOTE-062^5^ | First-line | Fluorouracil or capecitabine plus placebo | Pembrolizumab monotherapy OR capecitabine or fluorouracil plus pembrolizumab | Locally advanced/unresectable or metastatic gastric or gastroesophageal adenocarcinoma | No |
| CheckMate 649^6^ | First-line | Capecitabine or fluorouracil plus oxaliplatin | Capecitabine or fluorouracil, oxaliplatin and nivolumab OR nivolumab plus ipilumumab | Unresectable advanced or metastatic gastric, gastro-oesophageal junction, or oesophageal adenocarcinoma, | Yes |
| TAGS^7^ | Third-line (or later line) | Placebo | Trifluridine/tipiracil | Non-resectable metastatic gastro-esophageal junction or gastric adenocarcinoma | No |

^1^Bang et al., Lancet, 2010

^2^Waddell et al. 2013

^3^Fuchs et al, Lancet, 2014

^4^Wilke et al., Lancet Oncology, 2014

^5^Shitara et al. JAMA Oncology, 2020

^6^Janjigian et al. Lancet, 2021

^7^Shitara et al. Lancet Oncology, 2018

**Supplementary table 2. Cumulative survival rates after one, three and five years**

|  | Number of patients | One year | Three years | Five years |
| --- | --- | --- | --- | --- |
| **All patients diagnosed with EAC, GEJC or GAC** | 7390 | 20.8% | 3.8% | 2.2% |
| Esophageal | 3346 | 22.2% | 3.8% | 2.1% |
| Gastroesophageal junction or cardia | 1246 | 23.2% | 4.8% | 2.2% |
| Gastric | 2798 | 17.9% | 3.5% | 2.3% |
| **Patients with HER2 negative carcinoma receiving first-line therapy** | 1932 | 29.3% | 3.9% | 2.2% |
| Esophageal | 797 | 31.0% | 4.3% | 2.2% |
| Gastroesophageal junction or cardia | 391 | 31.3% | 4.6% | 2.6% |
| Gastric | 744 | 26.4% | 3.1% | 1.8% |
| **Patients with HER2 negative carcinoma receiving first-line CapOx/FOLFOX** | 1415 | 29.9% | 3.6% | 2.2% |
| Esophageal | 594 | 31.4% | 3.4% | 1.9% |
| Gastroesophageal junction or cardia | 282 | 31.1% | 4.1% | 1.8% |
| Gastric | 539 | 27.5% | 3.6% | 2.8% |
| **Patients with HER2 positive carcinoma receiving first-line trastuzumab containing therapy** | 520 | 46.4% | 12.4% | 5.9% |
| Esophageal | 305 | 47.1% | 10.7% | 8.2% |
| Gastroesophageal junction or cardia | 111 | 53.2% | 17.0% | 5.7% |
| Gastric | 104 | 37.2% | 12.5% | 2.7% |

**Supplementary table 3. Median time to treatment failure (TTF) from start of first-line in patients with esophageal, gastroesophageal junction or gastric cancer.** Follow-up was available for patients with primary diagnosis in 2015-2017 who received first-line systemic treatment.

|  | Number of patients | Median TTF (months) | p-value |
| --- | --- | --- | --- |
| **Patients with HER2 negative carcinoma receiving first-line therapy** |  |  | 0.02 |
| Esophageal | 329 | 5.0 |  |
| Gastroesophageal junction or cardia | 175 | 5.3 |  |
| Gastric | 319 | 4.3 |  |
| **Patients with HER2 positive carcinoma receiving first-line trastuzumab containing therapy** |  |  | 0.33 |
| Esophageal | 118 | 6.0 |  |
| Gastroesophageal junction or cardia | 35 | 6.5 |  |
| Gastric | 51 | 8.0 |  |

**Supplementary table 4. Multivariable Cox regression of overall survival in patients with esophageal, gastroesophageal junction or gastric cancer.** The multivariable regression model is stratified by performance status, cT stage, cN stage and HER2 status, as these variables did not meet the proportional hazards assumption.

|  | Number of patients | Events | Median OS (months) | Univariable regression, HR (95% CI) | Multivariable regression, HR (95% CI) |
| --- | --- | --- | --- | --- | --- |
| **Primary tumor location** |  |  |  |  |  |
| Esophageal | 3346 | 3192 | 5.1 | Reference |  |
| Gastroesophageal junction or cardia | 1246 | 1178 | 5.2 | 0.97 (0.91-1.04) | 1.07 (0.99-1.15) |
| Gastric | 2798 | 2682 | 4.0 | 1.14 (1.09-1.20) | 1.05 (0.98-1.13) |
| **Sex** |  |  |  |  |  |
| Male | 5423 | 5178 | 4.8 | Reference | Reference |
| Female | 1967 | 1874 | 4.2 | 1.10 (1.04-1.16) | 1.01 (0.95-1.07) |
| **Age** | - | - | - | 1.02 (1.02-1.02) | 1.00 (1.00-1.00) |
| **Number of comorbidities** |  |  |  |  |  |
| 0 | 3521 | 3328 | 5.4 | Reference | Reference |
| 1 | 2238 | 2150 | 4.3 | 1.17 (1.11-1.23) | 1.03 (0.97-1.09) |
| ≥2 | 1310 | 1268 | 3.8 | 1.33 (1.25-1.42) | 0.99 (0.92-1.06) |
| Unknown | 321 | 306 | 4.8 | 1.02 (0.90-1.14) | 0.89 (0.78-1.01) |
| **Lauren classification** |  |  |  |  |  |
| Intestinal | 2985 | 2823 | 5.3 | Reference | Reference |
| Diffuse | 2044 | 1977 | 3.9 | 1.27 (1.20-1.35) | 1.27 (1.18-1.36) |
| Mixed | 224 | 207 | 5.5 | 1.04 (0.91-1.20) | 1.12 (0.96-1.30) |
| Indeterminate | 234 | 221 | 4.3 | 1.13 (0.98-1.30) | 1.12 (0.97-1.29) |
| Adenocarcinoma NOS | 1903 | 1824 | 4.4 | 1.14 (1.07-1.20) | 1.02 (0.96-1.09) |
| **Tumor differentiation** |  |  |  |  |  |
| Well/moderate | 1688 | 1580 | 6.3 | 0.78 (0.73-0.83) | 0.83 (0.77-0.89) |
| Poorly/undifferentiated | 2905 | 2786 | 4.3 | Reference | Reference |
| Unknown | 2797 | 2686 | 4.1 | 1.02 (0.97-1.08) | 0.94 (0.89-1.00) |
| **Distant metastatic sites** |  |  |  |  |  |
| 0 | 233 | 203 | 7.5 | 0.71 (0.61-0.81) | 0.90 (0.74-1.09) |
| 1 | 4050 | 3827 | 5.6 | Reference | Reference |
| ≥2 | 3107 | 3022 | 3.4 | 1.39 (1.33-1.46) | 1.15 (1.05-1.26) |
| **Non-regional lymph nodes metastases** | 2934 | 2804 | 4.4 | 1.07 (1.02-1.12) | 1.15 (1.07-1.24) |
| **Lung metastases** | 1261 | 1220 | 3.9 | 1.14 (1.07-1.22) | 1.52 (1.41-1.63) |
| **Liver metastases** | 3298 | 3178 | 4 | 1.15 (1.10-1.21) | 1.02 (0.94-1.10) |
| **Peritoneal metastases** | 2140 | 2058 | 3.8 | 1.20 (1.14-1.26) | 1.42 (1.31-1.54) |
| **Bone metastases** | 1104 | 1082 | 3.4 | 1.36 (1.27-1.45) | 1.4 (1.29-1.52) |
| **Other metastatic sites** | 1028 | 1012 | 2.8 | 1.49 (1.40-1.59) | 1.32 (1.21-1.43) |
| **Type of treatment** |  |  |  |  |  |
| Palliative resection | 298 | 226 | 14.8 | 0.16 (0.14-0.19) | 0.18 (0.15-0.21) |
| Chemoradiotherapy | 110 | 96 | 12.7 | 0.20 (0.16-0.24) | 0.26 (0.21-0.32) |
| Systemic therapy | 3011 | 2783 | 9.2 | 0.30 (0.28-0.31) | 0.28 (0.27-0.30) |
| Best supportive care | 3971 | 3947 | 2.2 | Reference | Reference |


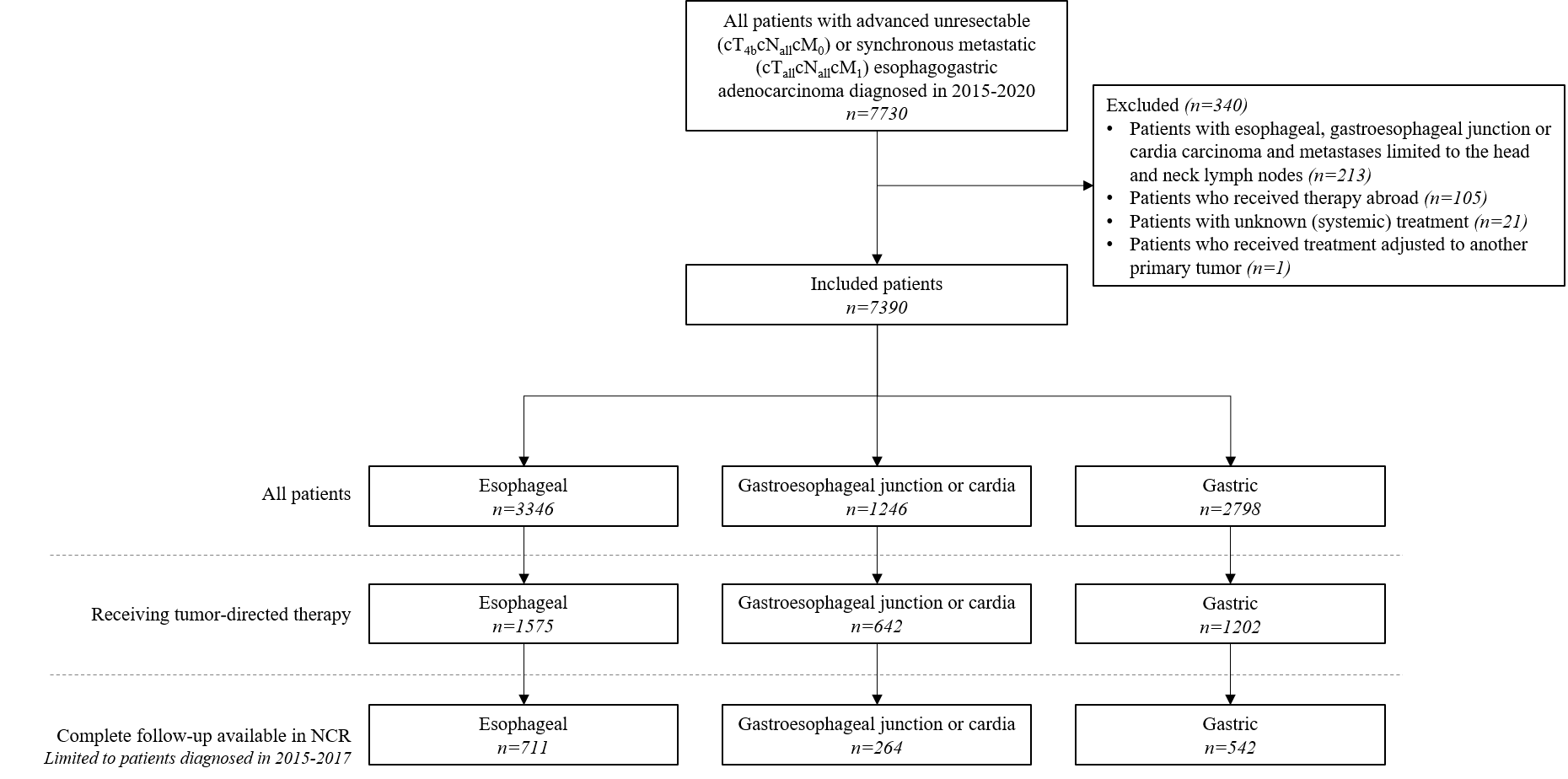


**Supplementary figure 1. Flowchart of patient selection.** Patients with esophageal, gastroesophageal junction or cardia carcinoma and metastases limited to the non-regional lymph nodes in the head and neck region (n=213) were excluded as these patients could have been eligible for treatment with curative intent. Patients who received treatment abroad (n=105) and patients for whom (systemic) treatment was unknown (n=21) were excluded. Finally, one patient was excluded because the treatment plan was adjusted for a secondary tumor. Complete follow-up (including data on treatment failure and second-line systemic therapy) was registered in the second half of 2019 for all patients diagnosed in 2015-2017 and who received tumor-directed therapy. Follow-up regarding vital status was available for all patients and complete until February 1, 2022.

**Supplementary figure 2.** **Type of second-line systemic treatment in patients with esophageal, gastroesophageal junction or gastric adenocarcinoma for patients in whom follow-up was available.** Follow-up was available for all patients who received first-line systemic treatment and primary diagnosis in 2015-2017 (n=1324). Second-line systemic treatment was administered in 393 out of the 1324 patients. EAC: esophageal adenocarcinoma; GEJC: gastroesophageal junction adenocarcinoma; GAC: gastric adenocarcinoma.


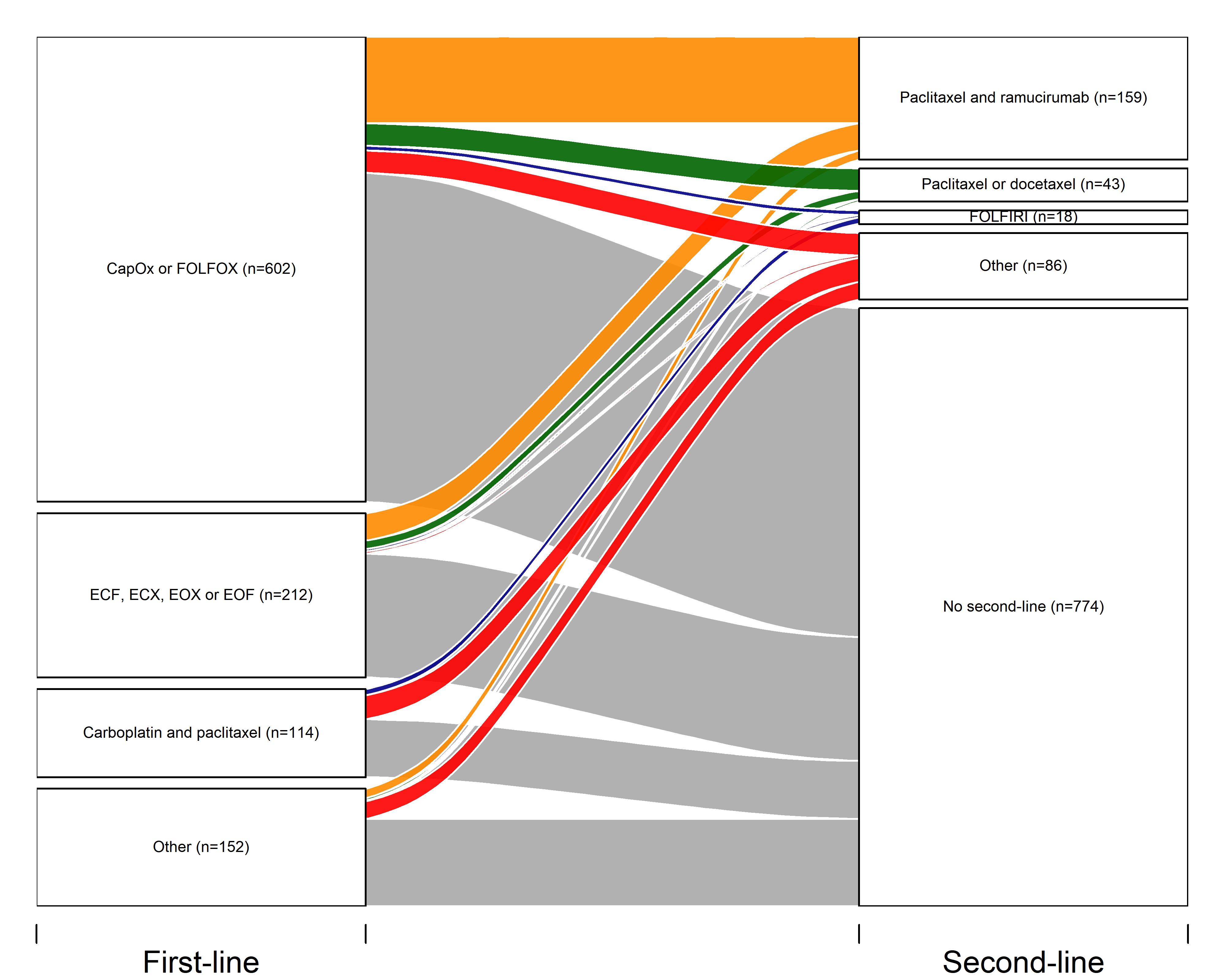


**Supplementary figure 3. Alluvial diagram of first- and second-line treatment in patients with esophageal, gastroesophageal or gastric cancer with a HER2-negative tumor or unknown HER2 status.** Specific regimens were limited to the three most common in first- and second-line. CapOx: capecitabine and oxaliplatin; FOLFOX: 5-FU and oxaliplatin; ECF: epirubicin, cisplatin and 5-FU; ECX: epirubicin, cisplatin and capecitabine; EOX: epirubicin, oxaliplatin and capecitabine; EOF: epirubicin, oxaliplatin and 5-FU; FOLFIRI: 5-FU and irinotecan.

**A**

**B**

**Supplementary figure 4. Overall survival of patients with esophageal, gastroesophageal junction or gastric cancer receiving second-line treatment (A), and receiving second-line paclitaxel and ramucirumab (B).** mOS: median overall survival, IQR: interquartile range.


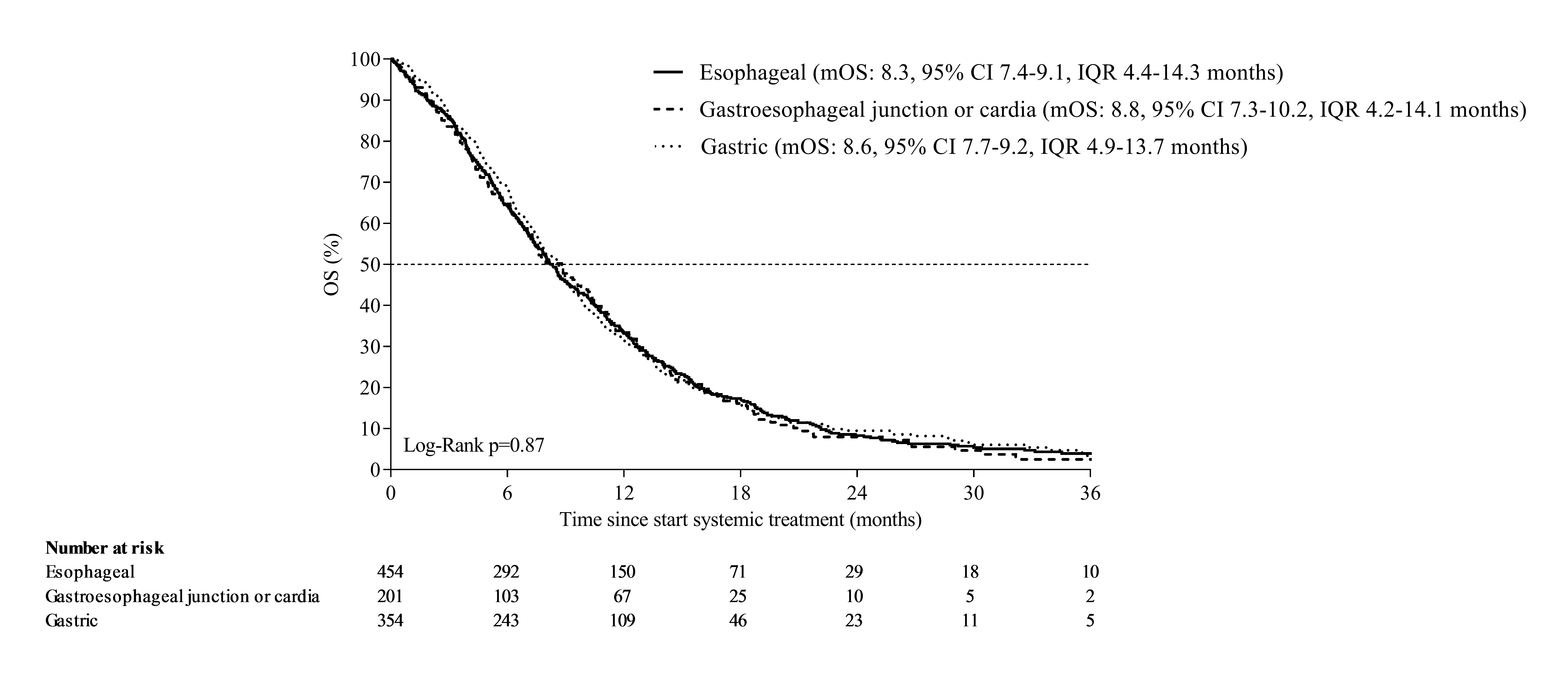


**Supplementary figure 5. Overall survival of patients with esophageal, gastroesophageal junction or gastric HER2-negative cancer with a performance status of 0-1 receiving first-line capecitabine or 5-FU with oxaliplatin.** mOS: median overall survival, IQR: interquartile range.
